# Patient trust in the health system, Internet information searching and the patient-provider relationship

**DOI:** 10.3389/fmed.2025.1665927

**Published:** 2025-11-20

**Authors:** Arch G. Mainous, Rachel Liu-Galvin, Barbara Durden, Lu Yin, Aaron A. Saguil

**Affiliations:** 1 Department of Community Health and Family Medicine, University of Florida, Gainesville, FL, United States; 2 Department of Health Services Research, Management and Policy, University of Florida, Gainesville, FL, United States

**Keywords:** trust, patient-provider interaction, survey, Internet, USA

## Abstract

**Importance:**

A positive patient-provider relationship is critical to the delivery of quality healthcare.

**Objectives:**

To examine the association between patients’ level of trust in the healthcare system, Internet information searching and the patient-provider relationship.

**Methods:**

Analysis of adult patients aged ≥ 18 in the US nationally representative Health Information National Trends Survey (HINTS 7-2024) (unweighted *n* = 2,510; weighted *n* is > 87 million). The patient-provider relationship was assessed focusing on discussing health information found on the Internet with their provider: (1) whether the provider was open to the discussion, (2) whether the provider was respectful, and (3) whether the interaction worsened. Respondents were asked how much they trust the healthcare system.

**Results:**

The proportion of patients with high trust in the healthcare system was 31%. Patients with low trust (17%) were more likely than those with high trust (3%) to perceive that in visits with their healthcare provider, talking about information found on the Internet, the patient-provider interaction became worse (*p* < 0.01). In logistic regressions controlling for multiple covariates, low trust in the healthcare system was associated with an increased likelihood that in visits with their healthcare provider, talking about information found on the Internet, the patient-provider interaction became worse (OR 6.76; 95% CI 2.35, 19.44).

**Conclusion:**

Patients with low trust in the healthcare system are at greater risk of having less than optimal relationships with their healthcare provider due to discussions over health information found on the Internet.

## Introduction

A positive patient-provider relationship is critical to delivery of healthcare. A positive relationship has been shown to yield greater treatment adherence in terms of medication use and receipt of preventive services (1–4). Trust in the healthcare system and providers in general play a role in a positive relationship and adherence to healthcare recommendations (1, 3, 5).

There is decreasing trust in government health agencies and the information that they provide (6). Despite this, trust in physicians has remained high. It has been suggested that the trust that patients have in physicians may encourage patients to trust the information provided by generally acknowledged healthcare experts (7). People with low trust in an information source are likely to search for information from other sources. The Internet provides many opportunities for finding information that gives a different picture of reality (8, 9). However, as patient trust in the information provided by healthcare experts and the healthcare system drops, distrust of treatments recommended by healthcare experts and systems may ultimately contribute to searching the Internet for other information.

Patients may have difficulty distinguishing between more and less trustworthy sources of online information and inadequate coping with conflicting or contradictory health information (10). Further, it has been suggested that patients who find this information on the Internet and bring it to a doctor’s visit might end up challenging their clinicians’ authority, as well as endorsing untrustworthy information and that this could lead to an argument or conflict with them (11).

A recent report looked at social media and other resources that patients use to gather health information. What this report showed was that 38% of those aged 18–34 had disregarded their provider’s medical guidance in favor of using social media which is a 12-point increase from 2024 (12). With this, over 70% of respondents at all ages felt confident in their ability to tell good medical advice from bad and find trustworthy health information (12). Previous systematic reviews have looked at data from the years of the Covid-19 pandemic to see the patient-provider relationship in regard to trust in the healthcare system, but nothing has been done post pandemic (13).

Since it is unclear how trust in the healthcare system is affecting patient physician interactions in contemporary times, we undertook an investigation of a nationally representative survey of US adult patients, the Health Information National Trends Survey (HINTS) 7 from the National Cancer Institute (NCI) conducted in 2024. The objective of this study is to examine the association between patients’ level of trust in the healthcare system, Internet information searching and the patient-provider relationship. It is hypothesized that patients with lower trust in the healthcare system will report higher levels of disruption in their interactions with healthcare providers in discussing Internet gathered health information.

## Methods

We conducted an analysis of the National Cancer Institute’s Health Information National Trends Survey (HINTS) data, a nationwide and population-based survey [Survey Instruments | HINTS (cancer.gov)]. This study utilizes the 2024 HINTS 7 database. This de-identified data is freely available to the public from the National Cancer Institute and is considered “not human subjects.” This study was deemed exempt from review by the Institutional Review Board at the University of Florida. Individuals aged 18 or older in the civilian non-institutionalized population of the United States were included.

### Outcome variables

#### Patient-provider relationship in the context of discussing health information found on the Internet with their provider

The patient-provider relationship in the context of discussing health information found on the Internet with their provider was assessed using three separate binary outcome variables. These variables focus on capturing patient perceptions when discussing health information found on the Internet with their provider: (1) whether the provider was open to the discussion, (2) whether the provider was respectful, and (3) whether the interaction worsened. Each outcome was analyzed independently. These items follow from the literature on the potential impact of discussions of Internet acquired health information in a patient visit with a healthcare provider (10).

The first item was assessed based on the respondent’s agreement with the following statement “*In the past 12 months, when I talked with a healthcare professional about information I found on the Internet, they were open to talking with me*” (strongly disagree, somewhat disagree, somewhat agree, strongly agree). Responses were recoded into two levels, agree (strongly or somewhat agree) and disagree (strongly or somewhat disagree).

The second item was assessed based on the respondent’s agreement with the statement “*In the past 12 months, when I talked with a healthcare professional about information I found on the Internet, they respected what I had to say*” (strongly disagree, somewhat disagree, somewhat agree, strongly agree). Responses were again recoded into two levels, agree (strongly or somewhat agree) and disagree (strongly or somewhat disagree).

The third item was assessed based on the respondent’s agreement with the statement “*As a result of talking to my doctor, nurse, or other health professional about health information from the Internet, our interaction became…*” (a lot better; a little better; no impact; a little worse; or a lot worse). We recoded the initial response categories into the evaluative categories of worse (a little worse or a lot worse) or not worse (no impact, a little better, or a lot better) as a way to quantify conflicts in the relationship.

### Independent variable

#### Trust in the health care system

Because of previous studies showing the importance of trust in health care and health care agencies we evaluated a general measure of trust in the health care system (6, 7). Trust in the health care system was measured with the question: “*How much do you trust the health care system (e.g., hospitals, pharmacies, and other organizations involved in health care)?*” Response options focused on magnitude of trust (not at all; a little; some; and a lot*).* We evaluated trust with the four categories. We also recoded this variable theoretically into low trust (“not at all,” “a little” or “some”) and high trust (“a lot”).

### Covariates

We computed several other variables that we used as covariates in the analyses. These included the respondent’s age, race/ethnicity, education, and income. The respondent’s sex was not available in the database in accordance with the Presidential Executive Order of 20 January 2025, titled “Defending Women from Gender Ideology Extremism and Restoring Biological Truth to the Federal Government.”

In addition to these demographic characteristics, we examined several other important covariates. These included self-reported health status and political viewpoint. Past research shows that individuals with chronic conditions are more likely to search the Internet for health information than those without chronic conditions (14). In this analysis, we recoded self-reported health status into three categories: good (0 chronic conditions), fair (1–2 chronic conditions), and poor (3 or more chronic conditions). Similarly, political views significantly influence how people perceive, use, and trust online health information (15). We recoded political viewpoint into three categories: liberal (“very liberal,” “liberal,” “somewhat liberal”), conservative (“very conservative,” “conservative,” “somewhat conservative”) and moderate. We included a variable on digital health literacy. The question was “How much do you agree or disagree with the following statements: I have the skills to find the health information I need on the Internet.” We also included a variable that may be relevant to the perception of patient provider interaction in a discussion of a patient bringing forward information found on the Internet for discussion with the provider. The variable is “Have you ever experienced prejudice or been discriminated against when getting medical care?”

### Statistical analysis

Analyses were conducted using the “survey” package in R studio (version 4.5.0), which accounts for the complex survey design including stratification, clustering, and weighting. Sampling weights based on the 2024 HINTS 7 complex sample design were used in the statistical analysis. The survey package functions “svychisq” and “svyglm” were utilized to derive nationally representative estimates of non-institutionalized US population, chi-squared tests and logistic regression to assess the associations between trust in health care system, social media information and the patient-provider relationship.

To assess the association between trust in the healthcare system, and the patient–physician relationship, we first conducted bivariate analyses using Chi-Squared tests, followed by a series of logistic regression models. We began with unadjusted analyses and then conducted adjusted analyses controlling for potential confounding variables. The adjusted models controlled for age, race, income, education level, health status, and political affiliation.

## Results

The analysis was based on an unweighted sample of 2,510 individuals, representing a weighted population of 87,092,823. The population characteristics of adult patients aged 18 years or older are detailed in Table 1. The 2,510 number is the number of participants who have talked to the healthcare professionals about any kind of health information they found on the Internet in the past 12 months.

**TABLE 1:** Population estimates for demographic characteristics of patients aged ≥ 18 years, 2024 (unweighted *N* = 2C).

Factors	Population proportion (%)
Unweighted sample size	2,510
Weighted population size	87,092,823
**Age (%)**	
18–39 years	34
40–59 years	38
60–79 years	24
80 and above	4
**Race/ethnicity (%)**	
Non-Hispanic White	59
Non-Hispanic Black	10
Hispanic	14
Other	17
**Education (%)**	
Less than high school	4
High school and above	96
**Annual income (%)**	
Less than $20,000	14
$20,000–$90,999	35
$100,000 and above	51

Trust in the healthcare system indicated that 31% of patients had “a lot,” 46% had “some,” 17% had “a little,” and 6% had “not at all.” The proportion of patients with high trust was 31% and low trust was 69%. The unadjusted relationship between trust in the healthcare system and patient characteristics obtained from the Chi-Squared tests are shown in Table 2. Age was strongly associated with trust in the health care system with older individuals more likely to report high trust. In contrast, race/ethnicity, political viewpoint, and education were not significantly associated with trust in the health care system.

**TABLE 2:** The relationship between trust in the healthcare system and patient characteristics.

Trust in health care system				
Patient characteristics		High (%)	Low (%)	*P*
Age	18–39	60	40	< 0.001
	40–59	66	34	
	60–79	82	18	
	80+	92	8	
Race/ethnicity	Non-Hispanic White	73	27	0.06
	Non-Hispanic Black	69	31	
	Hispanic	64	36	
	Other	64	36	
Education	High school and above	70	30	0.32
	Less than high school	64	36	
Annual income	0–20 k	64	36	0.01
	20–100 k	76	24	
	100 k+	67	33	
Health status	Good	65	35	0.02
	Fair	73	27	
	Poor	66	34	
Political viewpoint	Liberal	76	24	0.08
	Conservative	66	34	
	Moderate	69	31	

The results of the Chi-Squared tests indicate that patients with low trust in the healthcare system are less likely to have positive relationships with providers as they discuss information found on the Internet. Specifically, only 75% of low-trust patients agreed that providers were open to talking about the information they found on the Internet in an interaction with a healthcare professional about information they found on the Internet, compared with 93% of high-trust patients (*p* < 0.001). Likewise, only 71% of low-trust patients agreed that providers respected what they had to say, compared with 94% of high-trust patients (*p* < 0.001). In contrast, 17% of low-trust patients reported that the interaction with their provider became worse after discussing the information found on the Internet, compared with just 3% of high-trust patients (*p* < 0.01).

Table 3 presents the unadjusted and adjusted logistic regression results for the relationship between trust in the healthcare system and the three outcomes representing conflict in the patient-provider relationship. Patients with low trust in the health care system were more likely to perceive that their provider was not open to discussing Internet-sourced information. Individuals with low trust also had higher odds of perceiving that their provider did not respect them during such discussions. Additionally, low trust was associated with higher odds of reporting that their interaction became worse after discussing Internet information with their provider. All of these associations between trust and potential conflict with their healthcare provider were significant in both the unadjusted and adjusted models.

**TABLE 3:** Logistic regression examining the association between trust in healthcare system and patient-perceived conflict in the patient-provider relationship.

	Patient-provider relationship					
	Patient perceives that the provider is not open to discussing information the patient found on the Internet		Patient perceives that the provider does not respect the patient in a discussion about information found on the Internet		Patient perceives that when the patient had a discussion about health information from the Internet, the interaction with the provider became worse	
	**Odds ratios (95% CI)**		**Odds ratios (95% CI)**		**Odds ratios (95% CI)**	
	Adjusted*	**Unadjusted**	Adjusted*	**Unadjusted**	Adjusted*	**Unadjusted**
Low trust	4.780 (2.031, 11.252)	4.283 (2.211, 8.295)	7.299 (2.903, 18.351)	6.602 (3.673, 11.866)	6.762 (2.352, 19.443)	6.383 (3.038, 13.412)

*The adjusted model controlled for age, race/ethnicity, education, annual income, health status, digital health literacy, perception of past discrimination in the healthcare system and political viewpoint. Respondent sex was not included because it was redacted in the US government dataset in compliance with the Executive Order “Defending Women from Gender Ideology Extremism and Restoring Biological Trust to the Federal Government.”

## Discussion

The results of this study show that patients with low trust in the healthcare system are more likely to perceive potential disruption in their relationship with their healthcare provider when discussing health information found online. The magnitude of this association across the three different outcomes we used to assess conflict in the patient-provider relationship was quite striking. These results have substantial implications for treatment adherence and use of preventive services. Although previous research has indicated that the general public has high trust in the health information provided by physicians, when examined in the context of interactions with healthcare providers, trust in the healthcare system, or more accurately a lack of trust, suggests that we may be on the cusp of a rising number of conflicts between patients and providers.

Given the strong association we observed between low trust in the health care system and an increased likelihood of reporting perceived disruption in the patient-provider relationship, it is important to consider why some patients may hold low levels of trust. A recent study found that low trust in public health agencies was associated with perceptions that health recommendations were both politically influenced and inconsistent (16). Such perceptions may also extend to recommendations from providers, meaning that when provider recommendations are inconsistent with health information found online, patients’ trust in providers and the health care system may decrease.

In this study, we observed that individuals with low trust in the health care system were more likely to report that their interactions with providers worsened after discussing online information. This finding points to a potential vicious cycle: low trust increases the likelihood of perceived conflict as indicated by the patient perceiving that the interaction with the provider worsened, and these difficult interactions may in turn further decrease trust in providers and the health care system. This dynamic presents both a challenge and an opportunity for providers, who must engage patients with varying levels of confidence in the health care system while fostering constructive, trust-building communication, particularly when discussing online information that may conflict with their own recommendations. Such interactions are important opportunities to prevent further decline in trust and to strengthen the patient-provider relationship.

The patient-provider relationship is a critical component to delivering quality healthcare and it can be particularly challenging in the context of patients finding information on the Internet and discussing the information in the visit. Patients look to the healthcare provider to interpret symptoms and test results (7). In a time where there is misinformation being disseminated it is important that the healthcare provider be an interpreter of information and a source of expertise in messaging (17). We were not able to determine if the information that the patient found on the Internet and discussed with the provider was accurate. It is hard to argue that searching information on the Internet by the patient is a negative behavior. When discussing information found on the Internet, perhaps the patients were collaborating with the provider in making sense of their conditions or treatment. As we look to the future of social media influence and its interaction with healthcare it’s important to be aware of these interactions.

As access to medical information on the Internet either through government sources, healthcare providers, or social media continues to grow the potential for disagreements between the patient and the healthcare provider continues to grow. As health information becomes increasingly accessible online, it is crucial that providers continue to encourage open, respectful discussions with patients about the information they encounter. Some patients may have limited access to healthcare providers or good sources of health information and move to the Internet as a way to overcome this lack of support. Patients who access the Internet for health information tend to be female, having some college or a college degree, and are middle class by income (18, 19). The types of information that they search for is related to wellness or a disease the patient may have (19, 20). Providers should proactively and non-judgmentally address patients’ questions and concerns, particularly when misinformation is present, or when online information conflicts with evidence-based recommendations. The use of motivational interviewing, active listening, as well as potentially getting further training on the use of skills similar to clinical mediators may be particularly useful (21).

A strength of our study was that by using the nationally representative 2024 HINTS 7 survey, our findings are generalizable to the US population. Another strength was our use of three outcome variables to comprehensively evaluate the patient-provider relationship when discussing health information found on the Internet. Also with this survey we were able to see how a person’s health affected their trust in health care as there were questions related to diagnoses that participants had. Another strength of this study is that we adjusted for a broad set of covariates that may influence both trust in the health care system and perceptions of the patient-provider relationship, including age, race/ethnicity, income, education, political viewpoint, digital health literacy, perceptions of past discrimination in health care, and health status, thus reducing the risk of confounding.

A limitation of this study was that since we were using a public use dataset we were limited to the questions that were included in the initial survey. Although it provides nationally representative data and the included questions did allow us to test the hypothesis of trust in the healthcare system and several measures of patient-provider relationship there are more questions that one could conceptualize that might have helped to really provide richness to the study which were not included. Moreover, it is unfortunate that the US government restricted the data set from including the sex of the respondent. An additional limitation was the use of self-reported data, which may be susceptible to recall bias or social desirability bias. Since trust and perceived conflict in the interaction are subjective, self-report is the most appropriate method for assessing these experiences. A third limitation is that we attempted to compute ordinal logistic regressions to account for the full range of the variables but were unable to based on the distribution of responses. Given the subtle variety of response options, we used the variables with the additional levels to capture more nuanced information and conducted an ordinal logistic regression. However, due to severe imbalance between levels, the optimizer within the regression could not find a solution, and the regression failed. Thus, results from ordinal logistic regressions are not presented.

## Conclusion

As we move forward into the future, physicians need to be aware of the influence that the Internet and social media has on their patients. A potential way that they can contribute to keeping trust high and adherence to the healthcare provider’s recommendations is by being understanding with their patients when they bring information to the table. This is something that will not be changing any time soon. Keeping the patient-provider relationship a strong and positive one is critical to delivery of quality health care.

## Data Availability

The original contributions presented in this study are included in this article/supplementary material, further inquiries can be directed to the corresponding author.
